# The Pharmacogenetics of Statin Therapy on Clinical Events: No Evidence that Genetic Variation Affects Statin Response on Myocardial Infarction

**DOI:** 10.3389/fphar.2021.679857

**Published:** 2022-01-05

**Authors:** Stella Trompet, Iris Postmus, Helen R. Warren, Raymond Noordam, Roelof A. J. Smit, Elizabeth Theusch, Xiaohui Li, Benoit Arsenault, Daniel I. Chasman, Graham A. Hitman, Patricia B. Munroe, Jerome I. Rotter, Bruce M. Psaty, Mark J. Caulfield, Ron M. Krauss, Adrienne L. Cupples, Wouter J. Jukema

**Affiliations:** ^1^ Department of Internal Medicine, Section of Gerontology and Geriatrics, Leiden University Medical Center, Leiden, Netherlands; ^2^ Department of Cardiology, Leiden University Medical Center, Leiden, Netherlands; ^3^ William Harvey Research Institute, Barts and The London School of Medicine, Queen Mary University of London, London, United Kingdom; ^4^ Barts NIHR Biomedical Research Unit, London, United Kingdom; ^5^ Department of Epidemiology, Erasmus MC - University Medical Center Rotterdam, Rotterdam, Netherlands; ^6^ Department of Clinical Epidemiology, Leiden University Medical Center, Leiden, Netherlands; ^7^ Department of Pediatrics, University of California, San Francisco, San Francisco, CA, United States; ^8^ Institute for Translational Genomics and Population Sciences, Los Angeles BioMedical Research Institute at Harbor-UCLA Medical Center, Torrance, CA, United States; ^9^ Centre de Recherche de l’Institut Universitaire de Cardiologie et de Pneumologie de Québec, Québec City, QC, Canada; ^10^ Department of Medicine, Faculty of Medicine, Université Laval, Québec City, QC, Canada; ^11^ Division of Preventive Medicine, Brigham and Women’s Hospital, Boston, MA, United States; ^12^ Harvard Medical School, Boston, MA, United States; ^13^ Blizard institute, Barts and the London School of Medicine and Dentistry, Queen Mary University of London, London, United Kingdom; ^14^ Cardiovascular Health Research Unit, Department of Medicine, University of Washington, Seattle, WA, United States; ^15^ Department of Epidemiology, University of Washington, Seattle, WA, United States; ^16^ Department of Health Services University of Washington, Seattle, WA, United States; ^17^ Department of Biostatistics, Boston University School of Public Health, Boston, MA, United States; ^18^ NHLBI Framingham Heart Study, Framingham, MA, United States; ^19^ Einthoven Laboratory for Experimental Vascular Medicine, Leiden University Medical Center, Leiden, Netherlands; ^20^ Netherlands Heart Institute, Utrecht, Netherlands

**Keywords:** pharmacogenetics, statins, GWAS, cardiovascular disease, myocardial infarction

## Abstract

**Background:** The pharmacogenetic effect on cardiovascular disease reduction in response to statin treatment has only been assessed in small studies. In a pharmacogenetic genome wide association study (GWAS) analysis within the Genomic Investigation of Statin Therapy (GIST) consortium, we investigated whether genetic variation was associated with the response of statins on cardiovascular disease risk reduction.

**Methods:** The investigated endpoint was incident myocardial infarction (MI) defined as coronary heart disease death and definite and suspect non-fatal MI. For imputed single nucleotide polymorphisms (SNPs), regression analysis was performed on expected allelic dosage and meta-analysed with a fixed-effects model, inverse variance weighted meta-analysis. All SNPs with *p*-values <5.0 × 10^−4^ in stage 1 GWAS meta-analysis were selected for further investigation in stage-2. As a secondary analysis, we extracted SNPs from the Stage-1 GWAS meta-analysis results based on predefined hypotheses to possibly modifying the effect of statin therapy on MI.

**Results:** In stage-1 meta-analysis (eight studies, *n* = 10,769, 4,212 cases), we observed no genome-wide significant results (*p* < 5.0 × 10^−8^). A total of 144 genetic variants were followed-up in the second stage (three studies, *n* = 1,525, 180 cases). In the combined meta-analysis, no genome-wide significant hits were identified. Moreover, none of the look-ups of SNPs known to be associated with either CHD or with statin response to cholesterol levels reached Bonferroni level of significance within our stage-1 meta-analysis.

**Conclusion:** This GWAS analysis did not provide evidence that genetic variation affects statin response on cardiovascular risk reduction. It does not appear likely that genetic testing for predicting effects of statins on clinical events will become a useful tool in clinical practice.

## Introduction

Therapy with statins, 3-hydroxy-3-methylglutaryl coenzyme A reductase inhibitors, is widely used for the primary and secondary prevention of cardiovascular disease (Cholesterol Treatment Trialists et al., 2015). Statin therapy reduces LDL-cholesterol concentrations by 30–50% and is accompanied by a 20–30% risk reduction in cardiovascular events (Cholesterol Treatment Trialists et al., 2015). Behaviours (e.g. treatment adherence), medical conditions, and genetics all contribute to the inter-individual variation in lipid lowering response to statin therapy.

In addition to many smaller pharmacogenetic studies, a large genome-wide association study (GWAS), which investigated the genetic variation associated with the LDL cholesterol lowering response of statins identified four independent loci mapped to the APOE, LPA, SORT1, SCLO1B1 genes (Postmus et al., 2014a). While many pharmacogenetic studies have investigated the effect of statins on LDL-cholesterol lowering response, the pharmacogenetic effect on cardiovascular risk reduction in response to statin treatment has been studied in small candidate gene studies with limited success (Peters et al., 2008; Peters et al., 2010a; Peters et al., 2010b; Peters et al., 2011; Leusink et al., 2014; Li et al., 2015). The decrease in risk of cardiovascular events driven by statin therapy is largely due to the LDL lowering, but could also be in part be attributable by other ancillary mechanisms such as inflammation, thrombosis and anti-platelet mechanisms (Oesterle et al., 2017). Therefore, candidate gene studies into lipid lowering pathways might not capture all main pharmacogenetic effects responsible for the differential response to statin therapy with respect to cardiovascular events (Leusink et al., 2016).

Previously, three GWAS analyses have been performed to investigate the pharmacogenetics effect of statins on cardiovascular disease reduction, however two of these studies were relatively small and did not yield any genetic variants associated with differential cardiovascular event reduction by statins (Shiffman et al., 2012; Postmus et al., 2014b). The GWAS of Wei et al. demonstrated that the *LPA* gene could have a role in the differential response of statin on coronary events (Wei et al., 2018), but did not provide replication of this finding. In a pharmacogenetic GWAS study within the Genomic Investigation of Statin Therapy (GIST) consortium, we aimed to investigate whether genetic variation was associated with the response of statins on cardiovascular disease reduction, in particular myocardial infarction (MI).

## Methods

### Design and Contributing Studies

This study was conducted within the GIST consortium, which includes data from 11 large studies.

We conducted a two-stage design approach, in order to follow-up any potential discovery findings within independent data. The data in stage one comprised two randomized controlled clinical trials (RCTs) (ASCOT UK and PROSPER) and six observational cohort studies (ARIC, ASCOT UK OBS, CHS, FHS, HVH, RS) including *n* = 10,769 participants (4212 cases and 6557 controls): 7,215 in RCTs (658 cases and 6557 controls) and 3,554 cases from observational studies. The data in stage two comprised three studies: ASCOT SC OBS and RCT, and MESA, with *n* = 1,525 (180 cases and 1345 controls). The details for all participating studies are in Supplementary Table S1 and Supplementary Note S1.

### Subjects

Only subjects of European descent were included. For RCTs all subjects using placebo and statin treatment were included in this analysis. For observational studies, incident MI cases after starting statin treatment were included as cases. Subjects with a previous MI event before statin treatment were eligible for inclusion. All participants gave written informed consent and the study was approved by all institutional ethics committees.

### Outcome Definition

The investigated endpoint was incident MI (fatal and non-fatal) defined as coronary heart disease death and definite and suspect non-fatal MI (cases). Case definitions per cohort are described in Supplementary Note S1. For the RCTs the controls were defined as subjects who did not experience incident MI during the follow-up period. For observational studies, the control group consisted of subjects without an incident MI after initiating statin treatment.

### Genotyping, GWAS Analysis and Statistical Models

Genotyping, quality control, data cleaning and HAPMAP imputation were performed independently in each study as outlined in Supplementary Table S2. All analyses were performed with the expected allelic dosages for the imputed single nucleotide polymorphisms (SNPs). Each study independently performed their GWAS on incident MI.

As both RCT and observational studies are included, we used two different statistical models to investigate the pharmacogenetic effect of statins on MI, both assuming the same underlying relation. For RCTs an additive genetic model was assumed and each SNP tested using a Cox-proportional hazards regression model with MI as the outcome, adjusted for statin use and including an interaction term of statin use and SNP. The main parameter of interest was the interaction term between statin use and the SNP allelic dosage.

For observational studies we used a case-only design, with incident MI cases included, where the outcome variable in the statistical model was a binary indicator variable for statin use according to whether or not the subject was taking any type of statin prior to the myocardial infarction event and with SNP dosage as the predictor. This was assessed with binary logistic regression.

Analyses were additionally adjusted for age-, sex- and study-specific covariates (for example, ancestry principal components or country).

### Quality Control and Meta-Analysis

Centrally, within each study, SNPs with MAF <1% or imputation quality <0.3 were excluded from the analysis. QQ-plots were assessed for each study to check for between-study differences (Supplementary Figure S1). The software package METAL was used for performing a fixed effects, inverse variance weighted meta-analysis (Willer et al., 2010). The interaction betas from the RCTs and the genetic variants association betas from the case only studies were meta-analysed.

To correct for possible residual population stratification, genomic control was applied to each study within METAL by adjusting for the genomic inflation factor prior to meta-analysis.

### Follow-up From Stage 1 to Stage 2

All SNPs with *p*-values <5.0 × 10^−4^ in the stage 1 GWAS meta-analysis were selected for further investigation in stage 2. A maximum of two SNPs per independent gene region were selected, based on the lowest *p*-value of statistical significance. A total of 144 SNPs, within 103 independent loci, were selected for follow-up in the second stage.

The studies in stage 2 provided the regression estimates for these follow-up SNPs, and these were meta-analysed together. Results from the stage 1 and stage 2 meta-analyses for the 144 follow-up SNPs were combined using a fixed-effects model, inverse variance weighted meta-analysis in METAL.

### Significance Criteria

A SNP would be declared significant in stage 1 data alone if it reached the genome-wide statistical significance threshold of *p* < 5 × 10^−8^ in the meta-analysis. Next, a locus would also be considered significant if the top SNP at the locus reached genome-wide significance in the combined meta-analysis, with concordant direction of effect between stage 1 and stage 2.

### Additional Analysis

As a secondary analysis, we performed a look-up within our Stage 1 GWAS meta-analysis results for SNPs of interest that would possibly be associated with a pharmacogenetic effect of statin therapy on myocardial infarction. SNPs significantly associated with coronary events and/or with LDL or HDL changes after statin treatment based on previous GWAS studies were considered SNPS of interest. First we performed a look-up of 23 SNPs which were known to have genome-wide significant association with coronary events from the CARDIOGRAM study (Schunkert et al., 2011; Nikpay et al., 2015) at the time of analysis. Secondly, we performed a look-up of five SNPs with known associations of pharmacogenetic effects of statin therapy on LDL lowering and HDL changes, based on the large GWAS studies in the GIST consortium (Postmus et al., 2014a; Postmus et al., 2016). For each look-up analysis, to control for multiple testing, we applied a Bonferroni correction, using a *p*-value threshold for statistical significance of 0.05/28 = *p*= 0.0018.

## Results

**FIGURE 1:**
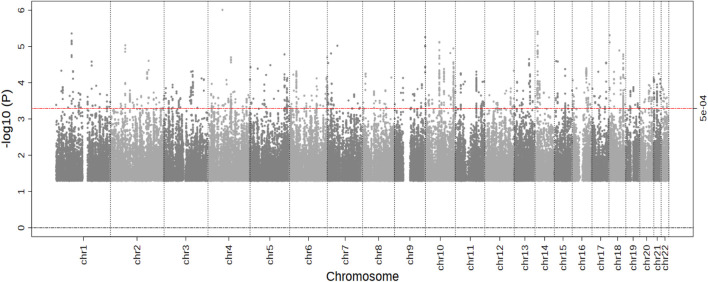
Results of the Stage 1 GWAS meta-analysis. Manhattan plot presenting the log10 *p*-values from the stage 1 meta-analysis (*n* = 10,769) on myocardial infarction risk after statin treatment. *p* values were generated using cox-proportional or logistic regression analysis. The blue line represents a *p*-value of 5.0 × 10^−4^. No SNPs reached the significance threshold of 5.0 × 10^−8^.

In the stage 1 meta-analysis (eight studies, 10,769 individuals, 4,212 cases), we observed no genome-wide significant results (*p* < 5.0 × 10^−8^) (Figure 1). The top signal was for rs4241669, mapping to *EPHA5* (beta, se: 0.330, 0.067) with a *p*-value of 9.8 × 10^−7^. The beta can be interpreted as an estimate of the interaction between statin use and the SNP allelic dosage on incident MI risk. A total of 144 genetic variants with *p*-value <5.0 × 10^−4^ were followed-up in the second stage. In the combined meta-analysis of stage 1 and stage 2, again no genome wide significant results were observed (Table 1), hence our overall results do not show any genetic variants with evidence of association with differential risk reduction in MI in response to statins. In Table 1 we present results for the top five genetic loci with *p* < 5.0 × 10^−6^). None of the top loci are near to known genes involved in pathways related to lipids or cardiovascular mechanisms.

**TABLE 1:** Main results for top five loci in combined meta-analysis of Stage 1 and Stage 2.

CHR	Position (hg18^a^)	Lead SNP	Gene	Coding allele	Non-coding allele	Frequency coding allele	Phase	*N*	beta	SE	*p*-value
4	66292902	rs4241669	*EPHA5*	C	T	0.234	Stage 1	10,769	0.330	0.067	9.77 × 10^−07^
							Combined	12,294	0.323	0.066	1.04 × 10^−06^
14	30821790	rs2181420	*HECTD1*	C	T	0.402	Stage 1	10,769	−0.277	0.060	3.91 × 10^−06^
							Combined	12,294	−0.260	0.059	9.13 × 10^−06^
18	2384986	rs7242734	*METTL4*	G	T	0.127	Stage 1	10,769	0.451	0.099	4.88 × 10^−06^
							Combined	12,294	0.440	0.096	4.53 × 10^−06^
1	71152038	rs11209716	*PTGER*	C	T	0.621	Stage 1	10,769	0.276	0.061	6.87 × 10^−06^
							Combined	12,294	0.241	0.060	6.10 × 10^−05^
9	138251691	rs7849585	*QSOX2*	G	T	0.330	Stage 1	10,769	−0.254	0.063	5.13 × 10^−05^
							Combined	12,294	−0.255	0.061	3.06 × 10^−05^

Gene: mapping of the SNP rsnumber based on UCSC genome browser.

a Imputation has been performed with Hapmap imputation.

Based on the look-up of SNPs known to be associated with either coronary events or with LDL cholesterol response to statin treatment (Table 2), a coronary event associated SNP at the *SORT 1* locus (rs599839) was most strongly associated with statin MI response, as was a SNP at this locus for the statin LDL response (rs646776). However none of the SNPs reached a Bonferroni level of significance within our stage 1 meta-analysis for statin response to MI risk (all *p* > 0.0018).

**TABLE 2:** Look-up of the most significant CARDIOGRAM on coronary events and previous top GIST loci with LDL and HDL response to statins within the Stage 1 meta-analysis results of this study.

Study	CHR	SNP	Gene	Coding allele	*N*	Beta	SE	*p*-value
CARDIOGRAM								
	1	rs11206510	*PCSK9*	C	10,769	0.1358	0.0906	0.1339
	1	rs599839	*SORT1*	G	10,769	0.1746	0.0743	0.0188
	1	rs17114036	*PPAP2B*	G	10,769	−0.0015	0.1035	0.9884
	2	rs6725887	*WDR12*	C	10,769	0.0391	0.0836	0.6401
	3	rs2306374	*MRAS*	C	10,769	0.0119	0.0792	0.8806
	6	rs12526453	*PHACTR1*	G	10,769	0.0440	0.0647	0.4965
	6	rs17609940	*ANKS1A*	G	10,769	−0.1318	0.0718	0.0664
	6	rs12190287	*TCF21*	G	10,769	−0.0737	0.074	0.3193
	7	rs11556924	*ZC3HC1*	C	10,769	−0.1637	0.0691	0.0178
	9	rs4977574	*CDKN2A/B*	G	10,769	−0.0699	0.0577	0.2257
	9	rs579459	*ABO*	C	10,769	−0.0108	0.0781	0.8900
	10	rs1746048	*CXCL12*	C	10,769	0.0043	0.0949	0.9639
	10	rs12413409	*CYP17A/CNNM2*	G	8,730	−0.0779	0.1077	0.4695
	11	rs964184	*ZNF259/APOcluster*	G	10,769	−0.1253	0.0912	0.1695
	12	rs3184504	*SH2B3*	C	10,769	0.0025	0.0579	0.9656
	13	rs4773144	*COL4A1/2*	G	9,555	0.00966	0.0774	0.2120
	14	rs2895811	*HHIPL1*	C	10,769	−0.0754	0.0584	0.1967
	15	rs3825807	*ADAMTS7*	G	10,769	−0.0659	0.0616	0.2847
	17	rs216172	*SMG6*	G	10,493	0.0611	0.0661	0.3553
	17	rs12936587	*RASD1*	G	10,769	0.1243	0.0601	0.0386
	17	rs46522	*UBE2Z/GIP*	C	10,769	0.0928	0.0592	0.1171
	19	rs1122608	*LDLR*	G	10,769	−0.0735	0.0683	0.2819
	21	rs9985601	*MRPS6*	C	10,769	0.0796	0.0895	0.3738
GIST LDL response to statins								
	1	rs646776	*SORT1*	C	10,769	0.1824	0.0732	0.0127
	6	rs10455872	*LPA*	G	7,018	−0.0188	0.1929	0.9224
	12	rs2900478	*APOE*	T	10,769	−0.0531	0.0785	0.4988
	19	rs445925	*SCLO1B1*	G	10,230	0.0263	0.1187	0.8247
GIST HDL response to statins								
	16	rs247616	*CETP*	c	10,769	−0.0541	0.0633	0.3927

## Discussion

In this genome-wide association study we did not identify any genetic variants with significant evidence of association between inter-individual variation to statin therapy and differential risk reduction for MI. Furthermore, we did not identify significant genetic effects on MI risk with statin treatment for any known CHD-associated variants or any known variants associated with statin LDL response.

There have been three previous pharmacogenetic GWAS studies of the modification of clinical benefit by statins (Shiffman et al., 2012; Postmus et al., 2014b; Wei et al., 2018). The first study by Shiffman *et al* reported an association in the *DNAJC5B* gene, although this was not genome-wide significant (Shiffman et al., 2012), and the result has never been replicated. Like our study, the other GWAS analysis by Postmus et al. found no significant pharmacogenetic associations (Postmus et al., 2014b). Both studies, however, were not sufficiently large by current GWAS standards to detect genome-wide significant associations with small effects. The third study by Wei et al. found that variants within the *LPA* gene were significantly associated with residual cardiovascular risk in statin users, (Wei et al., 2018), but our GWAS study did not replicate that result (*p* = 0.9224).

Our study has some limitations. Although it is the largest pharmacogenetic GWAS analysis of clinical cardiovascular response to statins to date, the number of cases (*n* = 4,392) is still relatively small. Leusink et al. calculated that to reach genome wide significance with an interaction odds ratio of 1.1, 15,000 MI cases would be required (Leusink et al., 2016). Knowing that small effects in large samples would not be relevant for clinical practice, we hypothesized that our study may still have sufficient statistical power to detect any potential relevant variants with larger effects sizes, if they were to exist. Another limitation could be that we included subjects from both randomized controlled trials and observational studies, and this could have generated some noise and therefore less precision.

In conclusion, this meta-analysis of GWASs, the largest one performed up until now investigating the pharmacogenetics of statin therapy on clinical events, did not provide any evidence that genetic variation affects statin response on coronary outcomes. Taken together with results of previous studies it does not appear likely that genetic testing for predicting effects of statins on clinical events can be a useful tool in clinical practice.

## Data Availability

The datasets presented in this study can be found in online repositories. The names of the repository/repositories and accession number(s) can be found below: https://www.ncbi.nlm.nih.gov/gap/, phs000930.v10.p1.
