# Supplementary material for: The Pharmacogenetics of Statin Therapy on Clinical Events: No Evidence that Genetic Variation Affects Statin Response on Myocardial Infarction
Source: Front Pharmacol. 2022 Jan 5;12:679857. doi: 10.3389/fphar.2021.679857 (PMC8769168; doi:10.3389/fphar.2021.679857)
Supplement: Supplementary file 1 [file DataSheet2.docx]

Supplementary Note 1 - Participating studies

*Atherosclerosis Risk in Communities (ARIC) study*

The ARIC study is an ongoing population-based cohort of 15,792 predominantly Caucasian and African-American males and females aged 45-64 years at baseline and selected using probability sampling from four United States communities (Forsyth County NC, Jackson MS, suburban Minneapolis MN, and Washington County MD) ^12^. Participants were recruited in 1987-1989 to examine cardiovascular and pulmonary disease, patterns of medical care, and disease variation over time. Standardized physical examinations and interviewer-administered questionnaires were conducted at baseline (1987-1989), three triennial follow-up examinations (1990-1998), and a fifth exam (2011-2013). Eligible participants for this effort were from the NC, MN, and MD field centers, as only Caucasian participants were examined in this analysis and the MS center only recruited African American participants. The Affymetrix 6.0 genotype array was used to genotype n=669,450 SNPs that passed quality control (sample call rate ≥ 0.95; SNP call rate ≥ 0.90; SNP MAF filter ≥ 0.01, HWE p-value filter ≥ 10^-5^). SNPs were imputed based on the HAPMAP build 36 with MACH v1.16 and analyses were performed using ProbABEL.

*Anglo-Scandinavian cardiac Outcomes Trial (ASCOT)*

Of 19,342 hypertensive patients (40–79 years of age with at least three other cardiovascular risk factors) who were randomized to one of two antihypertensive regimens in ASCOT, 10,305 with non-fasting TC concentrations of 6.5 mmol/l or less (measured at the non-fasting screening visit) had been randomly assigned additional atorvastatin 10 mg or placebo. These patients formed the lipid-lowering arm (LLA) of the study. For this genome-wide study only a proportion of United Kingdom, Irish, Sweden, Norway, Finland and Denmark consented to participate. For GWAS data, the available samples were genotyped separately, first for the UK and Irish GWAS (ASCOT-UK), and subsequently for the Scandinavian GWAS (ASCOT-SC). Within GIST analyses, ASCOT-UK was used within the discovery stage and ASCOT-SC within the replication. In both the GWAS resources there were two subpopulations from ASCOT included. The first subpopulation used the RCT data (ASCOT-RCT) and included individuals randomized to 10 mg atorvastatin in whom pre-treatment HDL-C was measured at the (fasting) randomization visit and on-treatment HDL-C was calculated as the simple average of measures at the (fasting) visits 6 months and 12 months post-randomization. Following the end of the randomization phase, there was an observational period. The second subpopulation used this observational data (ASCOT-OBS) and included all individuals not originally randomized to 10 mg atorvastatin (i.e., those randomized to placebo and those not eligible for the LLA) who were subsequently prescribed atorvastatin 10 mg during follow-up. Myocardial infarction was defined by Coronary Heart Disease death and non-fatal Myocardial infarction (incl. Silent).

*Cardiovascular Health Study (CHS)*

The CHS is a population-based cohort study of risk factors for CHD and stroke in adults ≥65 years conducted across four field centers ^18^. The original predominantly Caucasian cohort of 5,201 persons was recruited in 1989-1990 from random samples of the Medicare eligibility lists; subsequently, in 1992-1993, an additional predominantly African-American cohort of 687 persons was enrolled for a total sample of 5,888. DNA was extracted from blood samples drawn on all participants at their baseline examination. In 2007-2008, genotyping was performed at the General Clinical Research Center's Phenotyping/Genotyping Laboratory at Cedars-Sinai using the Illumina 370CNV BeadChip system on 3980 CHS participants who were free of CVD at baseline, consented to genetic testing, and had DNA available for genotyping. Because the other cohorts were predominantly white, the African American participants were excluded from this analysis. Thus, for this analysis, the study sample is limited to European ancestry participants who used statins during follow up with available genotype data as well as on- and off-treatment lipid measures.
In CHS, the following exclusions were applied to identify a final set of 306,655 autosomal SNPs: call rate < 97%, HWE P < 10-5, > 2 duplicate errors or Mendelian inconsistencies (for reference CEPH trios), heterozygote frequency = 0, SNP not found in HapMap. Imputation was performed using BIMBAM v0.99 with reference to HapMap CEU using release 22, build 36 using one round of imputations and the default expectation-maximization warm-ups and runs.

MI was defined by a combination of symptoms, cardiac enzyme and electrocardiographic changes.

1.  Ives DG, Fitzpatrick AL, Bild DE, et al.  Surveillance and ascertainment of cardiovascular events:  The Cardiovascular Health Study.  Ann Epidemiol 1995;5:278-285.

2.  Psaty BM, Delaney JA, Arnold AM, Curtis LH, Fitzpatrick AL, Heckbert SR, McKnight B, Ives D, Gottdiener JS, Kuller LH, Longstreth WT Jr.  The study of cardiovascular health outcomes in the era of claims data:  The Cardiovascular Health Study.  Circulation 2016;133:156-64.

*Framingham Heart Study (FHS)*

The methods for recruitment and clinical covariate collection have been described previously for the original Framingham Heart Study cohort (5,209 participants ascertained systematically from two-thirds of the households in the town of Framingham, MA, beginning in 1948)^20^, the Framingham Heart Study Offspring cohort (5,124 children of the original cohort, and spouses of those children, beginning in 1972)^21^, and the Third Generation cohort (4,095 children of the Offspring cohort, beginning in 2002)^22^. The current study was conducted in 263 participants recruited in the original cohort from Exam 23 through Exam 28, the Offspring Cohort from Exam 5 through Exam 8 (1991-2008) and the Third Generation from Exam 1 and Exam 2 (2003-2011). Genotyping was conducted for the SNP Health Association Resource (SHARe) project (<http://www.ncbi.nlm.nih.gov/projects/gap/cgi-bin/study.cgi?study_id=phs000007.v20.p8>) using the Affymetrix 500K mapping array (250K Nsp and 250K Sty arrays) and the Affymetrix 50K supplemental gene focused array on a total of 9,274 individuals from all three cohorts. BRLMM was used to call these data. To evaluate population substratification, we conducted principal component analyses using EIGENSTRAT ^23^ on the genotypes from 882 unrelated participants. We estimated the first 10 principal components and applied the loadings of these components to all genotyped participants. Genotyping resulted in 503,551 SNPs with successful call rate >95% and HWE P>1.0x10-6 in 8,481 individuals with call rate >97%. Imputation of 2,543,887 autosomal SNPs in HapMap release 22, CEU sample was conducted using the algorithm implemented in MACH (version 1.0.15). From a total of 534,982 genotyped autosomal SNPs in Framingham, 378,163 SNPs were used in imputation after filtering out 15,586 SNPs (HWE P<1.0x10^-6^), 64,511 SNPs (missingness >0.03), 45,361 SNPs (mishap P<1.0x10^-9^), 4,857 SNPs (>100 Mendel errors), 67,269 SNPs (frequency <0.01), 2 SNPs (due to strand issues upon merging data with HapMap), and a further 13,394 SNPs that were not present on HapMap. We used 200 biologically unrelated participants to estimate the parameters of the imputation model and subsequently applied the estimated parameters to obtain imputed SNPs for all 8,481 participants. Myocardial infarction was defined as a fatal or non-fatal incident MI based on electrocardiographic readings or elevated enzymes or autopsy evidence. The Framingham Heart Study, including genetic association studies of Framingham phenotypes, was approved by the institutional review boards of Boston University and the National Institutes of Health. All participants provided written informed consent.

*Heart and Vascular Health (HVH) Study*

HVH is a case-control study set in Group Health (GH), a large integrated health care system in Washington State, and is comprised of incident myocardial infarction (MI) and stroke cases with a shared common control group. All participants were GH members and aged 30-79 years. MI and stroke cases were identified from hospital discharge diagnosis codes and were validated by medical record review. Controls were a random sample of GH members frequency matched to MI cases on age (within decade), sex, treated hypertension, and calendar year of identification. The index date for controls was a computer-generated random date within the calendar year for which they had been selected. For MI cases, the index date was the date of admission for the first acute MI. Participants were excluded if they were recent enrollees at GHC, had a history of prior MI or stroke, or if the incident event was a complication of a procedure or surgery. Methods for the study have been described previously. ^24-26^
Eligibility and risk factor information were collected by trained medical record abstractors from a review of the GH medical record using only data available prior to the index date and through a telephone interview. Medication use was ascertained using computerized GH pharmacy records. A venous blood sample was collected from all consenting subjects, and DNA was extracted from white blood cells using standard procedures.
Genotyping was performed at the General Clinical Research Center's Phenotyping/Genotyping Laboratory at Cedars-Sinai using the Illumina 370CNV BeadChip system. Genotypes were called using the Illumina BeadStudio software. Samples were excluded from analysis for sex mismatch or call rate < 95%. The following exclusions were applied to identify a final set of 301,321 autosomal SNPs: call rate < 97%, HWE P < 10-5, > 2 duplicate errors or Mendelian inconsistencies (for reference CEPH trios), heterozygote frequency = 0, SNP not found in HapMap, inconsistencies across genotyping batches. Imputation was performed using BIMBAM with reference to HapMap CEU using release 22, build 36 using one round of imputations and the default expectation-maximization warm-ups and runs.

*Multi-Ethnic Study of Atherosclerosis (MESA)*

The Multi-Ethnic Study of Atherosclerosis (MESA) is a study of the characteristics of subclinical cardiovascular disease (disease detected non-invasively before it has produced clinical signs and symptoms) and the risk factors that predict progression to clinically overt cardiovascular disease or progression of the subclinical disease. MESA researchers study a diverse, population-based sample of 6,814 asymptomatic men and women aged 45-84. Thirty-eight percent of the recruited participants were white, 28 percent African-American, 22 percent Hispanic, and 12 percent Asian, predominantly of Chinese descent ^27^. Participants were recruited from six field centers across the United States and followed-up three times with an average time period of follow-up of 2 years between each visit. Data from four visits (exam1 to exam4) was used for the analysis. Subjects on statin treatment at the time point of follow-up visit and off treatment at the previous visit were qualified for inclusion. Phenotype (lipids measures before and after statin treatment) and genotype data were available for 360 Caucasian subjects. The tenets of the Declaration of Helsinki were followed and institutional review board approval was granted at all MESA sites. Written informed consent was obtained from each participant.
Genotyping was performed using the Affymetrix Genome-Wide Human SNP Array 6.0. IMPUTE version 2.1.0 was used to perform imputation for the MESA Caucasian participants (chromosomes 1-22) using HapMap Phase I and II - CEU as the reference panel (release #24 - NCBI Build 36 (dbSNP b126)). SNPs with MAF less than 0.02 or HWE p value less than 0.001 were removed from the analysis.

*PROspective Study of Pravastatin in the Elderly at Risk (PROSPER)*

All data come from the PROspective Study of Pravastatin in the Elderly at Risk (PROSPER). A detailed description of the study has been published elsewhere ^7, 8^. PROSPER was a prospective multicenter randomized placebo-controlled trial to assess whether treatment with pravastatin diminishes the risk of major vascular events in elderly. Between December 1997 and May 1999, we screened and enrolled subjects in Scotland (Glasgow), Ireland (Cork), and the Netherlands (Leiden). Men and women aged 70-82 years were recruited if they had pre-existing vascular disease or increased risk of such disease because of smoking, hypertension, or diabetes. A total number of 5,804 subjects were randomly assigned to pravastatin or placebo. A large number of prospective tests were performed including Biobank tests and cognitive function measurements.
A whole genome wide screening has been performed in the sequential PHASE project with the use of the Illumina 660K beadchip ^9^. Of 5,763 subjects DNA was available for genotyping. Genotyping was performed with the Illumina 660K beadchip, after QC (call rate <95%) 5,244 subjects and 557,192 SNPs were left for analysis. These SNPs were imputed to 2.5 million SNPs based on the HAPMAP built 36 with MACH imputation software. Myocardial infarction was defined by coronary heart disease death and/or fatal or non-fatal myocardial infarction.

*Rotterdam study*

The Rotterdam Study is a prospective population-based cohort study of chronic diseases in the elderly population. From 1990 to 1993, 7983 inhabitants of the suburb Ommoord in Rotterdam, the Netherlands, aged 55 years or older, entered the Rotterdam Study (RS-I), and have been continuously followed since then. Medication prescription data were obtained from all seven fully computerized pharmacies in the Ommoord suburb. These pharmacies dispense the prescriptions of more than 99% of all participants. Information on all filled prescriptions from January 1st 1991 until June 1st 2008 was available and included information on the product name of the drug, the Anatomical Therapeutical Chemical code, the amount dispensed, the prescribed dosage regimen and the date of dispensing. Furthermore, in 2000, an extended cohort was enrolled, the Rotterdam Study II (RS-II). 3011 inhabitants entered the study and have been continuously followed since then. Detailed information on design, objectives and methods of this study have been described before ^28, 29^. The Rotterdam Study has been approved by the medical ethics committee according to the Wet Bevolkingsonderzoek: ERGO (Population Screening Act: Rotterdam Study), executed by the Ministry of Health, Welfare and Sports of the Netherlands. All participants gave informed consent to participate in the study and to obtain information from treating physicians and pharmacy records, separately. At baseline examination of the Rotterdam Study, blood was taken from which genomic DNA was extracted, using the salting-out method ^30^. Microarray genotyping was performed in both Rotterdam Study cohorts, using the Infinium II HumanHap550K Genotyping BeadChip version 3 (Illumina Inc., San Diego, CA, USA). Genotyping procedures were followed according to the manufacturer’s protocols. Microarray genotyping procedures in the Rotterdam Study have been previously described ^31^.

*Treating to New Targets (TNT)*

The design of the TNT trial has been described in details elsewhere ^10^. In brief, 10 001 patients with stable coronary heart disease (CHD) and LDL-C levels <130 mg/dL (3.4 mmol/L) were randomly assigned to receive either 10 or 80 mg of atorvastatin per day and were followed up for a median of 4.9 years. After approval by the institutional review committee, informed consent for genetic analysis was sought on entry into the trial and 5966 DNA samples were obtained from consenting individuals. A subset was chosen for whole-genome analysis based on the cardiovascular events during the course of the trial and those individuals were matched 3:1 with controls based on age, gender, treatment arm, smoking, diabetes, hypertension, baseline lipid values, baseline glucose levels, and screening LDL-C. The Perlegen 322K array genotyping array was used to perform genome-wide genotyping. Samples and SNPs will call rate equal or under 98% were removed prior to the analyses. IMPUTE 2 (v. 2.1.0) and GTOOL (v.0.6.6) were used to impute additional SNPs which were analyzed for their association with LDL response with PLINK (v.1.07).
