# Supplementary material for: The Pharmacogenetics of Statin Therapy on Clinical Events: No Evidence that Genetic Variation Affects Statin Response on Myocardial Infarction
Source: Front Pharmacol. 2022 Jan 5;12:679857. doi: 10.3389/fphar.2021.679857 (PMC8769168; doi:10.3389/fphar.2021.679857)

**Supplementary Table 1:** Participating study characteristics

| **Study sample** | **Participants** | **Male,  N (%)** | **Age*, mean ± SD** | **Age*, range** | **Current Smokers, N (%)** | **Diabetes, N (%)** | **Hypertension,**  **N (%)** | **History of MI,**  **N (%)** |
| --- | --- | --- | --- | --- | --- | --- | --- | --- |
| **Randomized controlled trials** | ***N Overall*** |  |  |  |  |  |  |  |
| PROSPER  Cases  Controls | 5244  590  4654 | 2524 (48)  356 (60)  2168 (47) | 75.3 ± 3.3  75.7 ± 3.4  75.3 ± 3.3 | 70-83  70-83  70-83 | 1392 (27)  152 (26)  1240 (27) | 544 (10)  83 (14)  461 (10) | 3257 (62)  361 (61)  2896( 62) | 708 (14)  153 (26)  555 (12) |
| ASCOT UK RCT  Cases  Controls | 1971  68  1903 | 1754 (89)  60 (89)  1754 (89) | 64.1 ± 8.2  65.8 ± 8.4  64.0 ± 8.1 | 41-80  44-80  41-80 | 24.7  26.5  24.6 | 23.3  35.3  22.9 | 100  100  100 | 0  0  0 |
| **Observational studies** | ***N Overall*** |  |  |  |  |  |  |  |
| ARIC | 624 | 450 (72) | 61.1 **±** 6.0 | 45-74 | 136 (22) | 183 (29) | 334 (54) | 155 (25) |
| ASCOT UK OBS | 82 | 82.9 | 65.5 ± 7.3 | 46-78 | 24.4 | 32.9 | 100 | 0 |
| CHS | 484 | 222 (46) | 72.1 ± 4.64 | 65-88 | 47 (10) | 68 (14) | 295 (61) | 0 by design |
| FHS | 263 | 158 (60) | 69.3 ± 12.4 | 34-97 | 58 (22) | 66 (25) | 160 (61) | 30 (11) |
| HVH1 | 1228 | 566 (46) | 66.2 ± 9.40 | 32-80 | 218 (18) | 308 (25) | 940 (77) | 0 by design |
| HVH2 | 290 | 149 (51) | 65.0 ± 9.27 | 40-80 | 45 (16) | 75 (26) | 243 (84) | 0 by design |
| Rotterdam Study | 555 | 322 (58) | 77.0 ± 8.0 | 56-99 | 156 (28) | 72 (13) | 435 (78) | 81 (15) |
| **Second stage studies** | ***N Overall*** |  |  |  |  |  |  |  |
| ASCOT Scandinavia (OBS)  Ascot Scandinavia (RCT)  Cases  Controls | 44  1391  46  1345 | 84.1  78.5  87.0  78.2 | 60.8 ± 9.4  61.0 ± 8.8  63.9 ± 8.2  60.9 ± 8.8 | 44-78  40-80  43-79  40-80 | 54.5  44.0  47.8  43.9 | 27.3  23.4  30.4  23.1 | 100  100  100  100 | 0  0  0  0 |
| MESA | 90 | 55 (61) | 67.0 ± 10.4 | 45-84 | 54 (60.0) | 26 (28.9) | 74 (82) | 0 |
| *Age at DNA collection | | | | | | | |  |

**Supplementary Table 2:** Genotyping characteristics

| **Study sample** | **Participants** | **Genotyping platform** | **Calling algorithm** | **NCBI build** | **Imputation software** | **Analysis software** | **Exclusion criteria used** |
| --- | --- | --- | --- | --- | --- | --- | --- |
| **RCTs** | ***N Overall*** |  |  |  |  |  |  |
| PROSPER |  | Illumina Human 660_Quadv1 | Beadstudio | 36.22 | MACH v1.0 16 | ProbABEL | Sample call rate>=97.5%, SNP call rate >=98%, SNP MAF>0.01 |
| ASCOT UK RCT | 1971 | Illumina Human 370CNV | Beadstudio | 36.22 | MACH v1.0 16 | ProbABEL | Sample call rate <=95%, SNP call rate <=97%, HWE<=10E-7, relatedness |
| **Observational** | ***N Overall*** |  |  |  |  |  |  |
| ARIC | 624 | Affymetrix 6.0 | Birdseed | 36 | MACH v1.0.16 | ProbABEL | MAF <1%, call rate <95%, HWE<10E-5 |
| ASCOT UK OBS | 82 | Illumina Human 370CNV | Beadstudio | 36.22 | MACH v1.0 16 | ProbABEL | Sample call rate <=95%, SNP call rate <=97%, HWE<=10E-7, relatedness |
| CHS | 484 | Illumina Human 370CNV | BeadStudio | 36 | BIMBAM | R | Samples excluded for sex mismatch, discordance with prior genotyping, or call rate < 95% SNPs excluded for: call rate < 97%, HWE P < 10-5, > 2 duplicate errors or Mendelian inconsistencies (for reference CEPH trios), heterozygote frequency = 0, SNP not found in HapMap. |
| FHS |  | Affymetrix 250K Sty, 250K Nsp & MIPS 50K Gene Centric | BRLMM | 36.22 | MACH v1.0.15 | R 2.6.1 with lmekin | Sample call rate ≤ 97%, SNP call rate ≤ 95%, SNP >1000 Mendelian errors, Heterozygosity 5 SD from Mean (<25.758% or >29.958%) |
| HVH1 | 1228 | Illumina Human 370CNV | BeadStudio | 36 | BIMBAM | R | Samples excluded for sex mismatch or call rate < 95%. SNP exclusions: call rate < 97%, HWE P < 10^-5^, > 2 duplicate errors or Mendelian inconsistencies (for reference CEPH trios), heterozygote frequency = 0, SNP not found in HapMap, inconsistencies across genotyping batches. |
| HVH2 | 290 | Illumina Omni Express | GenomeStudio | 36 | MaCH | R | Samples excluded for sex mismatch or call rate < 95%. SNP exclusions: call rate < 97%, HWE P < 10^-5^, > 2 duplicate errors or Mendelian inconsistencies (for reference CEPH trios), heterozygote frequency = 0, SNP not found in HapMap, inconsistencies across genotyping batches. |
| Rotterdam Study | 555 | Illumina HumanHap 550K | Illumina GenomeStudio | 36.22 | MACH v1.0.15 | ProbABEL | Call rate <98%, HWE  P<10^-6^, or MAF<1% |
| **Second stage** |  |  |  |  |  |  |  |
| ASCOT Scandinavia | RCT: 1391  OBS: 44 | Illumina Human Omni Exome Express v8.1 | BeadStudio, followed by zCall | 37 | MACH v1.0.18 | R | GWAS: Exclude samples with: Discrepant sex, duplicate samples, relatedness at IBD pi_hat 0.25, <99% call- rate, heterozygosity +/- 3 SD from Mean, separately for MAF > 1% / < 1%. Excluded SNPs with: 0% MAF, <98% call rate, HWE p<10^-6^ |
| MESA | 90 | Affymetrix Genome-Wide Human SNP Array 6.0 | Affymetrix | 36.24 | IMPUTE v2.1.0 | SNPTEST | SNP call rate >=95%, Imputation information>0.30, SNP MAF>0.01 |


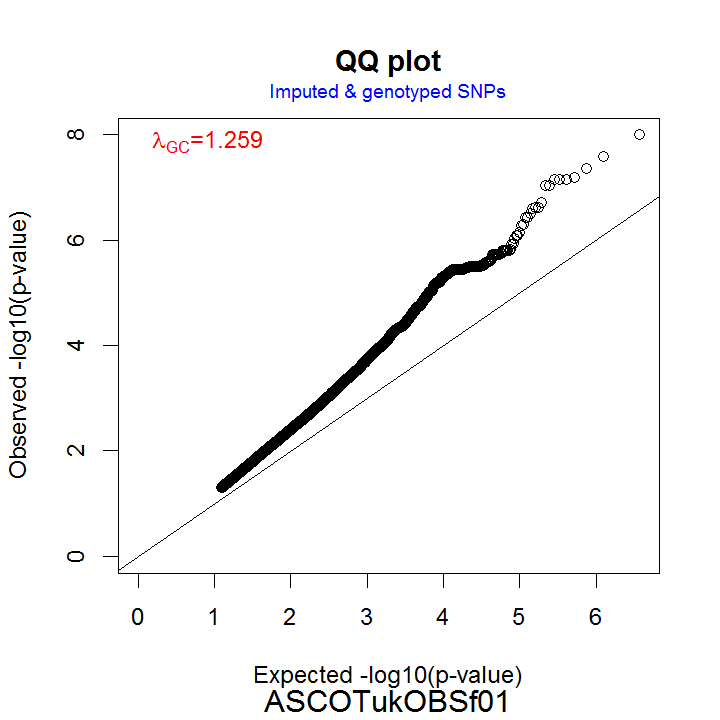

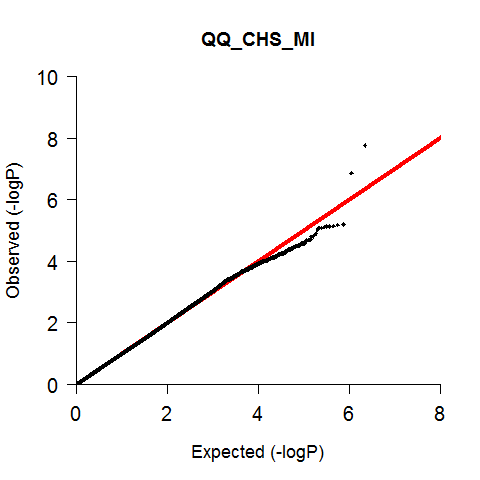

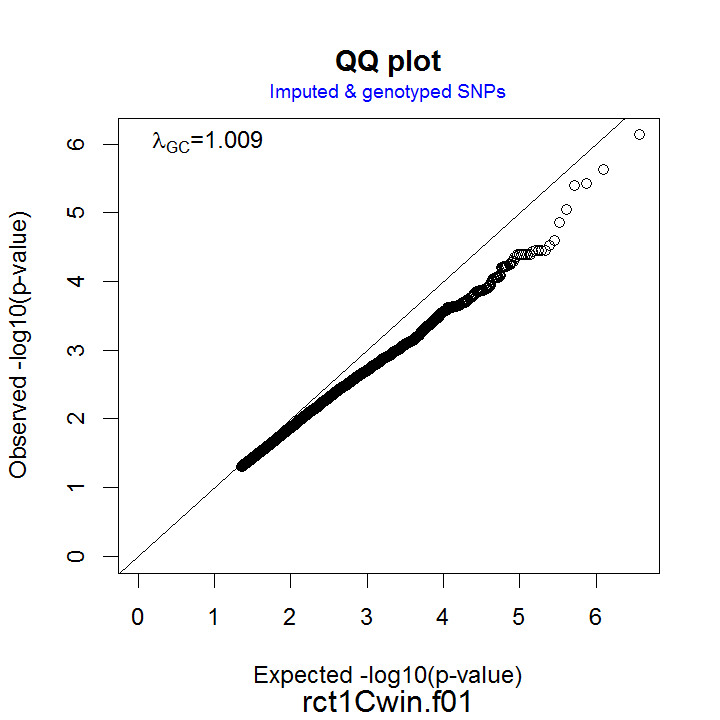

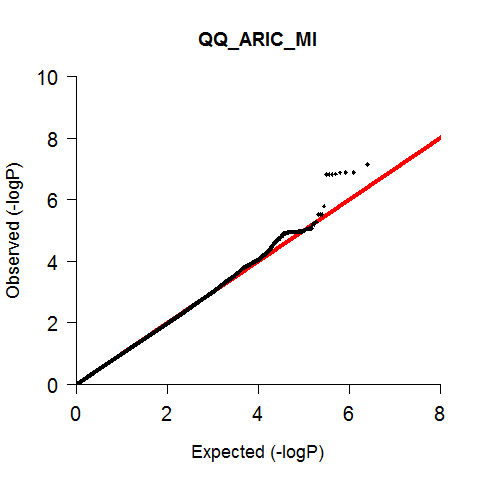
Supplementary Figure 1: Quantile-quantile plots of the expected versus observed -logP values for all studies participating in the first discovery stage.


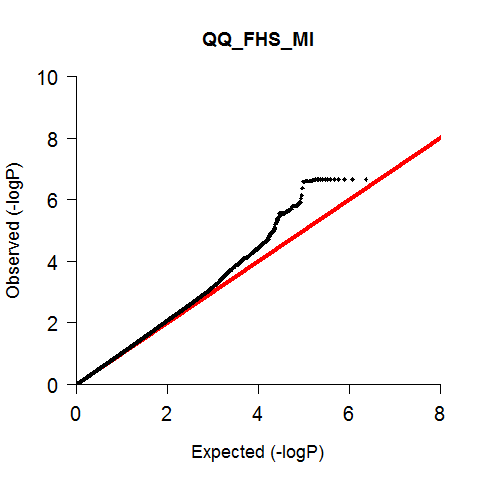

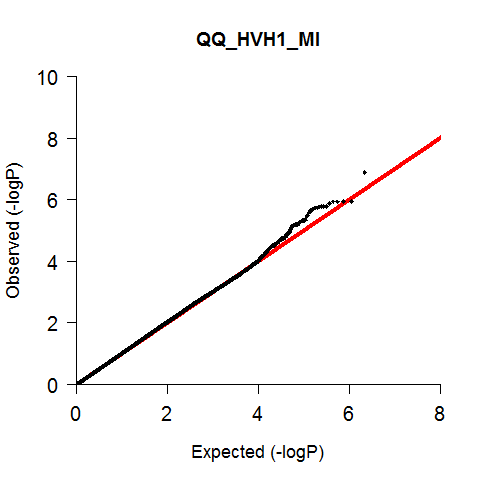

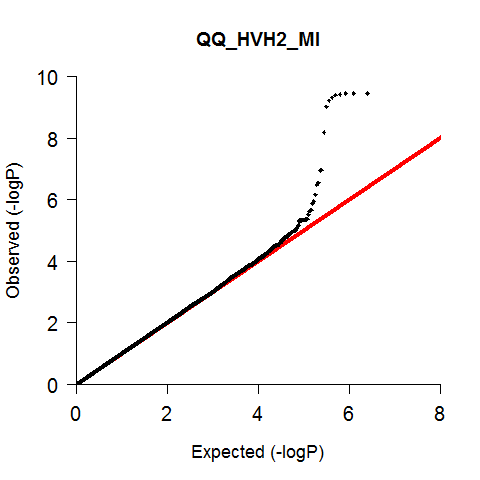

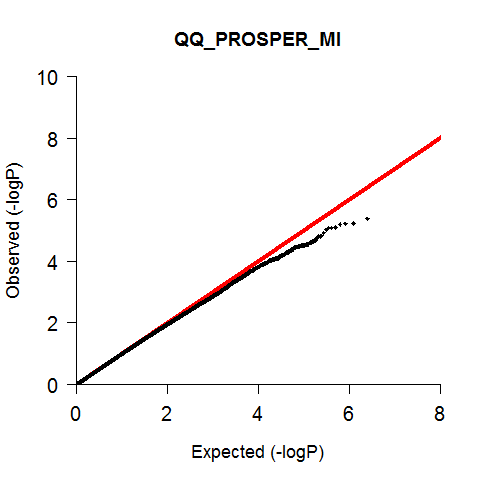


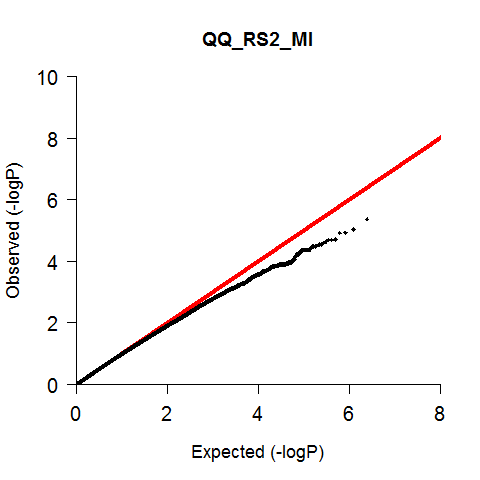

Supplement: Supplementary file 2 [file DataSheet1.docx]
